# Prevalence of masturbation and masturbation guilt and associations with partnered sex among married heterosexual Chinese males in an outpatient clinical setting: a retrospective single center study

**DOI:** 10.1186/s12610-025-00261-6

**Published:** 2025-04-22

**Authors:** Zheng Zhang, Nan Zhang

**Affiliations:** Department of Urology, The Second Affiliated Hospital of Zhejiang University School of Medicine, Hangzhou, Zhejiang 310009 China

**Keywords:** Masturbation, Masturbation guilt, Partnered sex, Married males, Frequency, Masturbation, Culpabilité de la masturbation, Relations sexuelles en couple, Hommes mariés, Fréquence

## Abstract

**Background:**

Solo masturbation is not an activity performed exclusively in single males but can also occur among married males, and is often associated with feelings of guilt. This study aimed to explore the prevalence of solo masturbation and associated masturbation guilt and the possible associated factors, including age, residence type (rural or urban), duration of marriage, parental status, income level, education level, body mass index, current smoking and drinking status, anxiety and depression status and their possible associations with the frequency of partnered sex among married heterosexual Chinese males.

**Materials and methods:**

A series of males attending our outpatient clinic were included and analyzed in the study. Approximately 71.2% (334/469) of these males had engaged in masturbation during the studying period, whereas 76.6% (256/334) of those who reported engaging in masturbation reported at least some sense of guilt.

**Results:**

Masturbation frequency was weakly positively associated with young age (OR 1.11) and education level (OR 1.24), whereas weak positive correlations were found between masturbation guilt and young age, anxiety and depression level (ORs ranged from 1.08 to 1.30). In addition, we found that the frequency of partnered sex was weakly positively associated with a masturbation frequency of less than once a month (adjusted OR 1.50) and once a month (adjusted OR 1.35). A weak positive correlation was observed between the frequency of partnered sex and a little sense of guilt (adjusted OR 1.60). In contrast, a weak negative association was observed between the frequency of partnered sex and a very big sense of guilt, with an adjusted OR of 1.67.

**Conclusions:**

In summary, in married heterosexual Chinese males, masturbation along with its associated guilt is a relatively frequent phenomenon. We obtained evidence supporting both compensatory and complementary relationships between masturbation, masturbation guilt and the frequency of partnered sex. Masturbation and its related guilt should receive more attention in clinical practice, given its high prevalence and possible relationship with partnered sex and couple relationships.

## Résumé

**Contexte:**

La masturbation en solo n’est pas une activité pratiquée exclusivement chez les hommes célibataires, mais peut également se produire chez les hommes mariés, et elle est souvent associée à des sentiments de culpabilité. Cette étude visait à explorer la prévalence de la masturbation solo et de la culpabilité associée à la masturbation, ainsi que les facteurs associés possibles, notamment: l’âge, le type de résidence (rurale ou urbaine), la durée du mariage, le statut parental, le niveau de revenu, le niveau d’éducation, l’indice de masse corporelle, le tabagisme et la consommation d’alcool actuels, l’anxiété et la dépression et leurs associations possibles avec la fréquence des rapports sexuels en couple chez les hommes chinois hétérosexuels mariés.

**Résultats:**

Une série d’hommes venant dans notre clinique en consultation externe ont été inclus dans l’étude. Environ 71,2% (334/469) de ces hommes s’étaient masturbés au cours de la période d’étude, et 76,6% (256/334) de ceux qui ont déclaré s’être masturbés ont dit avoir au moins un certain sentiment de culpabilité. La fréquence de la masturbation était faiblement associée positivement au jeune âge (RC 1,11) et au niveau d’éducation (OR 1,24), et de faibles corrélations positives ont été trouvées entre la culpabilité de la masturbation et le jeune âge, l’anxiété et le niveau de dépression (OR de 1,08 à 1,30). De plus, nous avons constaté que la fréquence des rapports sexuels avec une partenaire était faiblement associée positivement à une fréquence de masturbation de moins d’une fois par mois (OR ajusté 1,50) et d’une fois par mois (OR ajusté 1,35). Une faible corrélation positive a été observée entre la fréquence des rapports sexuels avec une partenaire et un faible sentiment de culpabilité (OR ajusté 1,60). En revanche, une faible association négative a été observée entre la fréquence des rapports sexuels avec une partenaire et un très grand sentiment de culpabilité (OR ajusté 1,67).

**Conclusions:**

Chez les hommes chinois hétérosexuels mariés, la masturbation et la culpabilité qui lui est associée constitue un phénomène relativement fréquent. Nous avons obtenu des preuves soutenant à la fois des relations compensatoires et complémentaires entre la masturbation, la culpabilité liée à la masturbation et la fréquence des rapports sexuels avec une partenaire. La masturbation et la culpabilité qui lui est associée devraient faire l’objet d’une plus grande attention dans la pratique clinique, compte tenu de sa prévalence élevée et de sa relation possible avec les relations sexuelles en couple et les relations de couple.

## Introduction

Masturbation is a common form of sexual activity across the lifespan and is usually regarded as a way to achieve one’s sexual pleasure. Masturbation refers to the self-stimulation of sexual organs, usually to the point of orgasm [1]. Although masturbation is a form of sexual behavior that can be performed alone or in the presence of others, it is more often examined within the context of solo sex and not in the context of partnered sex (solo masturbation). Solo masturbation is a common sexual practice among young and older adults but varies in reported frequency among different regions, cultures and genders. Data from a British study reported a masturbation frequency of 73% and 36.8% among males and females aged 16 to 44 years, respectively [2]. In the European male ageing study, that involving 3369 males from eight European regions, the overall incidence of masturbation was 17%, with males aged 40 to 49 years reporting the highest frequency (29%) and males over 70 reporting the lowest (7%) [3]. The reported masturbation frequency was 73.8% and 48.1% in males and females, respectively among American adolescents aged 14–17 years [4]. In a nationally representative sample (2828 respondents) from China, 13% of women and 35% of males reported engaging in masturbation in the preceding year. The incidence of ever engaging in masturbating in the last year among different age groups in urban China were 22% and 57% (20–29 years), 19% and 34% (30–39 years), 6% and 26% (40–49 years), 5% and 14% (50–59 years) for females and males, respectively, with overall incidences (20–59 years) of 15% and 35% for females and males, respectively. In contrast, the overall incidences in rural areas aged 20–59 years were 4% and 30% for females and males, respectively. In general, ever engaging in masturbation is more common among younger urban Chinese males [5].

Despite its high prevalence, masturbation has a long history of condemnation in different cultures, such as the Christian, Muslim and Jewish cultures [6]. Although masturbation is no longer considered as a negative activity and there is a reasonable literature on the beneficial effects of this behavior, including its individual, health and relational benefits [7], the cultural myths associated with masturbation may cause these males experiencing feelings of guilt or ashamed, which could them to experience distress or the desire to make changes or punish themselves, usually depending on their faith and cultural belief system. In Chinese culture, excessive semen loss associated with masturbation is believed to lead to Shen-Kui syndrome, which is characterized by several physical and psychological symptoms, including fatigue, weakness, musculoskeletal pain, dizziness and anxiety or depression [8]. In another culture in the Indian subcontinent, Dhat syndrome, for example, is associated with symptoms such as fatigue, weakness, loss of appetite, anxiety, guilt and fear of semen loss [9]. In a Korean study of males in the military, approximately 98% of the males had engaged in masturbation, and approximately 11% (132 of 1212) of these males had feelings of guilt [10]. In an Italian study, feelings of guilt were reported in 15.4% (274/1781) of these males who engaged in masturbation and were associated with more psychological disturbances and increased relational problems [11]. In addition, guilt after masturbation was reported by 8.4% of the included males and was found to be associated with a lower frequency of partner climax during partnered sex in another Italian study based on patients from a set of outpatient clinic [12]. However, a recent study based on Jewish single males revealed that masturbation guilt was positively related to psychological disturbance only at low levels of religiousness, whereas at high level of religiousness, this relationship was not found [6]. Gender differences also exist, usually normalizing masturbation among men while stigmatizing it among women [7]. Males are more likely to report masturbating than females are, as shown in a previous meta analysis, although the gender difference varies across countries, suggesting a sociocultural component of this gender difference. Gender differences in masturbation also exist in terms of sexual drive and pornography use: males are more likely to use erotic materials and have a stronger sex drive than women do [13].

The association between masturbation and sexual relationships has been described by compensatory or complementary models in previous studies. The former type of model prefers masturbation to replace partnered sex. The latter type of model favors that an increase in the practice of masturbation is associated with an increase in the frequency of partnered sex [14]. One study involving American males and females revealed that males who were satisfied with their sex lives were less likely to masturbate [15]. Another Spanish study revealed that current masturbation was negatively correlated with male orgasm satisfaction in partnered sex [16], supporting the compensatory model. Evidence from another study involving 1784 males supporting the complementary model revealed that males with greater desire to engage in partnered sex were more likely to report higher frequencies of masturbation in the past year [7]. Nevertheless, to simply conclude the relationship as either compensatory or complementary is not accurate, as it may work in a more complex way and depend on different individual and relationship characteristics, including gender, the presence of stable sexual partner [15], age, education level, income level [7] and psychological problems [17].

Masturbation is not an activity that occurs among single males but also occurs among married males with stable sexual partners. However, this phenomenon among married males with stable partners should receive additional attention since it has a potential correlation with couple relation and partnered sex. Nevertheless, this relationship among married Chinese males remains unclear and understudied. On the basis of these considerations, the present study aimed to: analyze the prevalence of solo masturbation and associated masturbation guilt among married heterosexual Chinese males; explore the possible background and individual factors associated with masturbation and masturbation guilt; analyze the possible factors associated with the frequency of partnered sex, and explore the possible mediating role of masturbation guilt in this relationship further.

## Materials and methods

### Study design and patients

From December 2021 to February 2023, a series of male patients attending our outpatient clinic who were seeking advice on sexual function or fertility issues for the first time were included if they met the following criteria: (a) were aged 18 years older, (b) were heterosexual and (c) were currently married. The study exclusion criteria were spinal cord injuries, pelvic or genitourinary trauma, penile deformation or penile fibrosis. All the data provided were collected as part of the routine clinical procedure. The present study was approved by the institution ethics committee (2023 0945) and informed consent was also obtained from the individuals. Primary interviews were conducted in the outpatient clinic using a self-defined questionnaire, and relevant data were collected. The entire interview process ranged from one half hour to one hour and was conducted by one doctor. No identifiable personal data (name, hospital identification number) were collected.

### Covariates

These covariates were considered as potential factors associated with sexual behaviors, especially masturbation and masturbation guilt. Masturbation frequency and its association with age have been described in previous studies [3, 5]. Education level is potentially associated with masturbation frequency [2] and masturbation guilt [18]. An association between masturbation frequency and regional differences has also been reported [18]. Income [19] and childbirth [20] were found to be related to the frequency of sexual activity. In addition, previous studies have yielded inconsistent findings on the relationships between health factors and masturbation [21]. Furthermore, masturbation guilt is associated with increased alcohol consumption as well as increased depression and anxiety symptoms [11]. On the basis of these considerations, data on the following covariates were collected: background factors, including age, residence type (rural or urban), duration of marriage, parental status, income level, education level, and health factors, including body mass index (BMI), current smoking and drinking status and anxiety and depression status.

### Measures

The frequency of solitary masturbation was assessed using a single item: “On average, how often have you masturbated by yourself in the past three months?” The response options were: never, Less than once a month, Once a month, more than once a month, more than once a week.

The feelings of guilt or discomfort after solo masturbation were also evaluated with a single question: “How do you feel after masturbation?” 0 = no guilt, 1 = with a little sense of guilt, 2 = with a big sense of guilty, 3 = with a very big sense of guilty.

For partnered sex evaluation, another item was used. We asked the participants, “On average, how often have you had sex with your partner (including oral sex, anal sex, vaginal sex, sex toy use and mutual masturbation with their partner) in the past three months?” The response options were: never, Less than once a month, Once a month, more than once a month, more than once a week.

Anxiety status was evaluated using the generalized anxiety disorder 7-item scale (GAD-7), which consists of 7 items. Each item is scored on a 4-point scale in relation to the past two weeks (0 = not at all; 1 = some of the time; 2 = more than half the time; 3 = nearly every day). The scale had shown good validity and reliability as an established anxiety measure (Cronbach’s alpha was 0.89) [22]. The GAD-7 score ranges from 0 to 21 points, with a higher score indicating a greater level of anxiety.

The depression status was assessed using patient health questionnaire-9 (PHQ-9), a self-rating inventory used to evaluate general depression status, which has good internal consistency (Cronbach’s alpha was 0.87) [23]. Each of the 9 items is rated on a four-point scale (0 = not at all; 1 = some of the time; 2 = more than half the time; 3 = nearly every day) in relation to the past two weeks. The total score ranges from 0 to 27 points, with a higher score indicating a greater level of depression.

### Statistical analysis

One-way Kolmogorov–Smirnov test was applied to check the normal distribution. Continuous variables that were normally distributed are presented as means ± standard deviations (SDs). Quantitative data that were nonnormally distributed are presented as medians (interquartile ranges). Logistic regression was used to identify potential significant factors related to masturbation and masturbation guilt. The associations between the frequency of partnered sex and potential factors including solo masturbation frequency and masturbation guilt were also examined by ordinal logistic regression analysis. The statistical analyses were performed using SPSS version 18.0 and a two-sided *p* value < 0.05 was considered to indicate statistical significance. Reliability was evaluated by internal consistency using McDonald’s ω, performed using SPSSAU, with a value of at least 0.70 considered to indicate acceptable reliability [24].

## Results

### Characteristics of the included males

Table 1 shows the descriptive statistics of the included males. A total of 512 males completed the questionnaire; 43 males were not included in the final analysis because of missing or invalid data. A total of 469 males were included in the final analysis. Most males lived in urban areas (382/469, 81.4%) and approximately 74.6% (350/469) of these males had at least one child. Over half (57.0%, 267/469) of the males had bachelor and graduate education level. The McDonald’s ω coefficients for the anxiety and depression scales were 0.78 and 0.82, respectively, indicating acceptable reliability.

**Table 1:** Characteristics of the included males

Characteristics	Value
Age (mean ± SD, years)	35.5 ± 8.0
BMI (mean ± SD, kg/m^2^)	21.5 ± 2.3
Residence, n(%)	
Urban	382(81.4%)
Rural	87(18.6%)
Duration of marriage (mean ± SD, years)	15.5 ± 5.8
Parental status, having child, n(%)	350(74.6%)
Income per month (mean ± SD, thousand yuan)	13.2 ± 4.4
Education level	
Senior high school or lower	202 (43.1%)
Bachelor and graduates	267 (56.9%)
Current smoking, n(%)	155 (33.0%)
Current drinking, n(%)	108 (23.0%)
GAD- 7(mean ± SD)	11.6 ± 4.5
PHQ- 9 (mean ± SD)	12.5 ± 3.8

Variables include: age (years, continuous), BMI (kg/m^2^, continuous), residence (urban/rural), parental status (yes/no), duration of marriage(years, continuous), income per month (thousand yuan, continuous), education (senior high school or lower/bachelor and graduates), current smoking status (yes or no), current drinking status (yes/no), anxiety score (continuous), depression score (continuous)

### Frequency of solo masturbation and masturbation guilt

Among the entire sample (469 males), 334 males (71.2%) reported that they had masturbated at least once in the past three months. In addition, among males who reported engaging in solo masturbation (334 males), approximately 27% (91/334, 27.0%) reported that they had masturbated less than once, whereas 55 males reported that they had masturbated once a month (55/334, 16.5%). A total of 81 males (24.0%, 81/334) reported that they had masturbated more than once a month during the past 3 months, whereas 107 (107/334, 32%) reported that they had masturbated more than once per week.

Masturbation guilt was reported by 256 males with an overall frequency of 76.6% (256/334) among males who reported engaging in masturbation, with 130 (130/334, 38.9%) males reporting a little sense of guilt, 62 (62/334, 18.6%) reporting a big sense of guilt, and 64 (64/334,19.1%) males reporting a very big sense of masturbatory guilt.

### Factors associated with masturbation and masturbation guilt

As expected, a weak positive correlation was found between masturbation and young age (adjusted OR (95% CI) = 1.11 (1.04–1.30), adjusting for BMI, residence type, parental status, duration of marriage, income level, education level, current smoking status, current drinking status, anxiety score, and depression score (OR 1.11, weak association) [25]. Additionally, as we expected, education level was weakly positively associated with masturbation frequency, with an adjusted OR of 1.24, adjusted for age, BMI, residence type, parental status, duration of marriage, income level, current smoking status, current drinking status, anxiety score, and depression score. Moreover, weak positive correlations were found between masturbation guilt and young age, the GAD-7 score and the PHQ-9 score (adjusted ORs ranged from 1.08 to 1.30; small effect size range, weak association) (Table 2).

**Table 2:** Factors associated with masturbation and masturbation guilt

Factors	Masturbation		Masturbation guilt	
	Adjusted OR (95% CI)	*P* value	Adjusted OR (95% CI)	*P* value
Age	1.11 (1.04–1.30)	0.01	1.08 (1.04–1.41)	0.02
BMI	0.88 (0.80–1.08)	0.64	1.01 (0.91–1.21)	0.98
Residence				
Urban	1.00	-	1.00	-
Rural	1.09(0.90–1.25)	0.30	0.87 (0.85–1.06)	0.18
Parental status	1.18 (0.81–1.38)	0.60	1.08 (0.91–1.15)	0.29
Duration of marriage	0.98 (0.90–1.15)	0.65	0.98 (0.88–1.12)	0.10
Income per month	1.09 (0.91–1.16)	0.41	0.85 (0.78–1.18)	0.64
Education level				
Senior high school or lower	1.00	-	1.00	-
Bachelor and graduates	1.24 (1.05–1.40)	0.01	1.13 (0.89–1.54)	0.19
Current smoking	0.88 (0.78–1.23)	0.90	0.91 (0.86–1.13)	0.84
Current drinking	0.98 (0.89–1.18)	0.43	0.89 (0.81–1.11)	0.17
Anxiety score (GAD- 7)	1.05 (0.88–1.15)	0.18	1.25 (1.02- 1.60)	0.04
Depression score (PHQ- 9)	0.92 (0.85–1.13)	0.16	1.30 (1.15–1.63)	0.02

Variables include: age (years, continuous), BMI (kg/m^2^, continuous), residence (urban/rural), parental status (yes/no), duration of marriage(years, continuous), income per month (thousand yuan, continuous), education (senior high school or lower/bachelor and graduates), current smoking status (yes or no), current drinking status (yes/no), anxiety score (continuous), depression score (continuous)

### Ordinal logistic regression analysis for the associations between the frequency of partnered sex and masturbation frequency, masturbation guilt

A significant association was observed between the frequency of partnered sex and age, masturbation frequency and masturbation guilt, with no other significant variables observed in the logistic regression analysis. Young age was weakly positively associated with partnered sex frequency (OR 1.05). In addition, we found that the frequency of partnered sex was weakly positively associated with a masturbation frequency of less than once a month and once a month (adjusted ORs ranging from 1.35 to 1.50, after adjusting for age, BMI, residence type, parental status, duration of marriage, income level, education level, current smoking status, current drinking status, anxiety score, depression score, and masturbation guilt). However, the association was not observed in males with a masturbation frequency of more than once a month or more than once a week compared with males who engaged in no solo masturbation.

Additionally, the frequency of partnered sex was significantly associated with masturbation guilt in our study. A weak positive correlation was observed between the frequency of partnered sex and a little sense of guilt (adjusted OR 1.60, after adjusting for age, BMI, residence type, parental status, duration of marriage, income level, education level, current smoking status, current drinking status, anxiety score, depression score, and masturbation frequency). In contrast, a weak but close to moderate negative association was observed between the frequency of partnered sex and a very big sense of guilt, with an adjusted OR of 1.67, after adjusting for age, BMI, residence type, parental status, duration of marriage, income level, education level, current smoking status, current drinking status, anxiety score, depression score and masturbation frequency. The results of the regression analysis are illustrated in Table 3.

**Table 3:** Ordinal logistic regression analysis for the associations between partnered sex frequency and masturbation frequency, masturbation guilt

	Adjusted OR	95% CI	p
Masturbation frequency			
Never	1.00	-	-
Less than once a month	1.50	1.22- 1.96	0.03
Once a month	1.35	1.12–1.84	0.02
More than once a month	0.89	0.76–1.20	0.94
More than once a week	1.13	0.74–1.59	0.56
Masturbation guilt			
No guilt	1.00	-	-
With a little sense of guilt	1.60	1.34- 1.89	0.04
With a big sense of guilt	1.15	0.85–1.37	0.55
With a very big sense of guilt	0.60	0.45–0.80	0.01
Age	1.05	1.00–1.20	0.02
BMI	1.10	0.77–1.89	0.66
Residence	0.92	0.75–1.32	0.38
Parental status	1.24	0.70–1.80	0.40
Duration of marriage	0.86	0.60–1.27	0.32
Income per month	1.15	0.88–1.46	0.76
Education level	0.84	0.74–1.30	0.50
Current smoking	1.21	0.50–1.99	0.41
Current drinking	0.86	0.55–1.33	0.34
Anxiety score (GAD- 7)	0.96	0.74–1.59	0.64
Depression score (PHQ- 9)	0.99	0.66–1.70	0.79

Variables include: age (years, continuous), BMI (kg/m^2^, continuous), residence (urban/rural), parental status (yes/no), duration of marriage(years, continuous), income per month (thousand yuan, continuous), education (senior high school or lower/bachelor and graduates), current smoking status (yes or no), current drinking status (yes/no), anxiety score (continuous), depression score (continuous), masturbation frequency, masturbation guilt

## Discussion

To our knowledge, this is the first report on the frequency of masturbation and masturbation guilt and their possible correlation with partnered sex in married heterosexual Chinese males. These results confirm that even among married Chinese males with stable partners, masturbation is a quite frequent behavior. We found that the majority of the subjects (71.2%) reported engaging in masturbation, and the percentage in this study was greater than that reported in a previous study [5], although the study population, data sources, and individual characteristics were different. Notably, the study was conducted during the coronavirus Disease 2019 (COVID-19) pandemic. The effect of COVID-19 on sexual function and sexual behavior has also been studied previously. Sexual activity, function and satisfaction significantly decreased in both males and females during the COVID-19 pandemic, whereas sexual activity including masturbation and the use of sex toys increased, as shown in a previous systematic review and meta analysis [26]. A similar finding was reported in another American study involving 1051 participants, in which there was a small decrease in partnered sex activities but a small increase in masturbation and pornography use among men [27]. The restriction of human activity, self-isolation, and fear of the possibility of transmitting the virus may partially explain the increased trend in the frequency of solo masturbation [28].

Most males who reported engaging in masturbation had at least some sense of guilt (76.6%), which was greater than that reported in previous Italian studies [11, 12]. The higher prevalence in this study was probably due to the long condemnation on masturbation in Chinese culture and the marital status of the included males, because of the shame or private nature associated with this behavior, especially among married males with stable relationships. In addition, financial burdens as well as emotional problems including stress, anxiety and depression during the COVID-19 pandemic have also been reported [28], which is in line with the findings of the present study. Nevertheless, during this period, the Chinese government took strict measures to control the spread of COVID-19, including social distancing, self-isolation and the implementation of severe acute respiratory syndrome-related coronavirus type 2 (SARS-CoV-2) vaccines. In addition, daily necessities and medical supplies were also provided in local communities. As part of China’s fight against COVID-19, the “health code” made great contributions in helping to track the flow state of people. People with “green” health codes were free to access public areas, including shopping markets and hospitals; thus their daily activities were basically unaffected.

Our data confirmed that solo masturbation was negatively related to age and positively associated with education level. Masturbation, as an education dependent behavior, is likely attributed to greater access to public debate, sex information, and sex education, which may help reduce fears and increase the willingness to report masturbation [2]. Our analyses also revealed that feelings of guilt about masturbation were more often observed in males, who presented features such as greater anxiety and depression tendencies and younger ages, although the presence of anxiety and depression symptoms did not correlate with masturbation behavior. These findings partially coincide with those of a previous study in which males with masturbation guilt may represent a more dysfunctional subpopulation showing more psychological and relational problems [12] and more features of poor couple relationships, hypogonadism, psychiatric disorders and depressive and anxiety symptoms [11], although further demographic and clinical parameters (social and couple disturbances, hormonal disorders) were not analyzed in the present study.

In this study, in terms of the interplay between partnered sex and masturbation as well as masturbation guilt, we found evidence for the complementary model. That is, the “mild” increase (less than once a month and once a month) in the frequency of solo masturbation and a low sense of guilt were weakly positively associated with a greater frequency of partnered sex. However, a greater frequency of masturbation was not associated with partnered sex. In addition, a very big sense of guilt was weakly but close to moderately negatively associated with the frequency of partnered sex, suggesting a compensatory model. Interestingly, in this case, there was a dual pattern, and masturbation guilt played a mediating role in this relationship; that is, a mild increase in solo masturbation and low sense of masturbation guilt were positively related to a greater frequency of partnered sex, whereas “excessive” or severe masturbation guilt was negatively associated with the frequency of partnered sex. We found that masturbation guilt was positively associated with anxiety and depression scores, with males with a greater degree of guilt showing a greater degree of anxiety and depression, which is in line with the findings of a previous study [12]. Although the relationship between psychological factors and sexual desire remains unclear, specific moods, including depression and anxiety, can promote sexual desire [29]. It may be that masturbation, or masturbation guilt associated with a negative emotional status, results in an increased sexual interest, and the transfer of these negative reactions creates a strong incentive to pursue sexual release and decreased anxiety and depression through orgasm, which is mainly obtained through solo masturbation [29]. Nevertheless, a high level of masturbation guilt may lead to severe depression and was associated with decreased sexual desire in previous case series [8, 30]. Although we found that proportional increases in anxiety and depression scores were associated with a greater degree of masturbation guilt, no significant associations were detected between anxiety and depression scores and the frequency of partnered sex. It may be assumed that a little sense of guilt or a low level of anxiety and depression was associated with more frequent partnered sex. However, males who had severe masturbation guilt and anxiety and depression status had a lower frequency of partnered sex. This may explain the nonsignificant associations between the proportional increases in anxiety and depression scores and the frequency of partnered sex. However, because of the complicated nature of sex, making a definite conclusion is not appropriate, although the present study sheds valuable light of on the characteristics of “pathological” masturbation (if exists) as well as the interplay between different variables and their associations with partnered sex. A possible objective method to define which degree of masturbation frequency, degree of guilt or combined as pathologic is warranted for future studies.

### Limitations

The results should be considered in light of several limitations. First, the inclusion of married heterosexual Chinese males seeking sexual/fertility related consultations at an outpatient clinic may introduce selection bias, thereby limiting the generalizability of our conclusions to broader populations, especially considering the background of the COVID-19 pandemic. Studies involving broader populations are needed, incorporating different religious and cultural backgrounds. Second, the cross-sectional study design inherently precludes causal relationships among masturbation frequency, masturbation guilt and the frequency of partnered sex. For instance, we cannot conclude that less masturbation and a low level of guilt contribute to partnered sex. It is also possible that partnered sex stimulates the demand for more sex, including masturbation. In addition, if people are not experiencing their desired amount of sex, masturbation may be an alternative; further clarification is needed if both masturbation and partnered sex increase. Conversely, it is also possible that people who are satisfied with their sex lives are less motivated to masturbate. This is in line with Regnerus, findings that subjective contentment with sexual life is associated with masturbation frequency [15], indicating that this relationship may be mediated by sexual satisfaction. We did not know the males’ desired frequency of sex, indicating that future studies investigating sexual desire along with the desired sex frequency are warranted. Moreover, the association of pornography with enhanced masturbatory responsiveness, but not partnered sex outcomes, has also been reported [31], indicating the possible role of pornography use in compensating for sexual dissatisfaction. This “weak” relationship between partnered sex and masturbation frequency found in this study indicates that more studies are needed to illustrate this relationship. In addition, we did not further explore the personal, family or social reasons why these males experienced masturbation guilt, which is highly important in intervention or treatment regimens. Third, there is no specific instrument to measure masturbation guilt and thus a scientific definition is lacking, establishing the onset and duration of this behavior is unlikely.

## Conclusions

To our knowledge, this is the first study investigating masturbation practices and their potential associations with partnered sex among married heterosexual Chinese males. Our findings indicate that masturbation is a prevalent behavior. In addition, 76.6% of the males engaged in masturbation had reported experiencing masturbation guilt. Moreover, masturbation guilt was found to be weakly positively associated with young age and GAD-7 and PHQ-9 scores. We also noted weak positive associations between the frequency of partnered sex and mild increase in masturbation frequency and a low sense of guilt, supporting the complementary model. In contrast, a weak negative correlation emerged between the frequency of partnered sex and a very high level of guilt, suggesting a compensatory model. However, given the limitations of the present study, future research with different cultural backgrounds incorporating more variables is needed.

## Data Availability

The data that support the findings of this study are available from the corresponding author upon reasonable request.

## Abbreviations

BMI: Body mass index
GAD-7: Anxiety disorder 7-item scale
PHQ-9: Patient health questionnaire-9
COVID-19: Coronavirus Disease 2019
SARS-CoV-2: Severe acute respiratory syndrome-related coronavirus type 2
